# Modeling of laser-induced breakdown spectroscopic data analysis by an automatic classifier

**DOI:** 10.1007/s41060-018-00172-y

**Published:** 2019-02-08

**Authors:** David D. Pokrajac, Poopalasingam Sivakumar, Yuriy Markushin, Daniela Milovic, Gary Holness, Jinjie Liu, Noureddine Melikechi, Mukti Rana

**Affiliations:** 1 Delaware State University, Dover, DE 19901 USA; 2 Southern Illinois University Carbondale, Carbondale, IL 62901 USA; 3 University of Nis, Aleksandra Medvedeva 14, 18000 Niš, Serbia; 4 University of Massachusetts Lowell, Lowell, MA 01854 USA

**Keywords:** Spectroscopy, Echelle, Laser-induced breakdown spectroscopy, Optimal classifier, Statistical learning theory.

## Abstract

Laser-induced breakdown spectroscopy (LIBS) is a multi-elemental and real-time analytical technique with simultaneous detection of all the elements in any type of sample matrix including solid, liquid, gas, and aerosol. LIBS produces vast amount of data which contains information on elemental composition of the material among others. Classification and discrimination of spectra produced during the LIBS process are crucial to analyze the elements for both qualitative and quantitative analysis. This work reports the design and modeling of optimal classifier for LIBS data classification and discrimination using the apparatus of statistical theory of detection. We analyzed the noise sources associated during the LIBS process and created a linear model of an echelle spectrograph system. We validated our model based on assumptions through statistical analysis of “dark signal” and laser-induced breakdown spectra from the database of National Institute of Science and Technology. The results obtained from our model suggested that the quadratic classifier provides optimal performance if the spectroscopy signal and noise can be considered Gaussian.

## Introduction

Laser-induced breakdown spectroscopy (LIBS) is a multi-elemental and real-time analytical technique with simultaneous detection of all the elements in any type of sample matrix including solid, liquid, gas, and aerosol [1]. In LIBS system, a pulsed laser—such as a Q-switched Nd:YAG, is focused onto the surface of the material to eject a tiny fraction of material (picograms to nanograms) from the surface of the object under investigation. By this process, forming short-lived, highly luminous plasma at the surface of the material is formed. Within this hot plasma, the ejected material is dissociated into excited ionic and atomic species. The excited ions and atoms emit characteristic optical radiation as they revert to lower energy states. Detection and spectral analysis of the optical radiation formed through this process is used to yield information on the elemental composition of the material which includes atomic composition of the compound.

During this excitation process, LIBS not only produces the data associated with the samples of interest but also from the unwanted sources like from the system. LIBS uses multiple spectrograph and synchronized charge-coupled device (CCD) spectral acquisition system to analyze the spectral data. For rapid analysis of heterogeneous materials, the acquisition cycle typically stores 1000 spectra for subsequent filtering and analysis. The incorporation of an effective data analysis methodology has been critical in achieving both accurate and reproducible results in the analysis of samples with the technology. LIBS produces vast amounts of data where one or multiple elements are falling almost at the same emission lines. Simultaneous elemental analysis is required to avoid sampling errors associated with the application of a destructive analysis technique LIBS uses for compositional determination of a heterogeneous material. Simultaneous elemental analysis also reduces the analysis time, thereby increasing sample throughput and efficiency of the whole system. To handle the huge amount of data produced by LIBS, the use of automatic classifier and discriminator for spectral analysis is necessary for accuracy, time saving, and increasing efficiency.

Automatic classification of spectroscopy data is a scientific and technical field where chemical molecules, compounds, and mixtures are distinguished based on their spectral signatures by means of computer algorithms  [2, 3]. Automatic classification has been attempted on various spectroscopy techniques: magnetic resonance [4], Fourier transform infrared spectroscopy (FTIR) [5], Raman spectroscopy [6], and LIBS data [7–11]. The utilized methods usually involve linear models (e.g., linear discriminant analysis [4, 5]) on amplitudes of some spectral components, selected by means of feature selection machine learning algorithms. Other publications describe utilization of principal component analysis of spectral components to reduce data dimensionality, followed by an instance-based machine learning algorithm that provides a linear or nonlinear model [6, 9–12]. While these approaches may perform well in practice, they are ad hoc and lack theoretical justification; more specifically, there is no assurance of their optimality from the point of view of statistical theory of detection.

In this work, we report the model and design of an optimal classifier for automatic classification and discrimination of LIBS data. We also use experimental data to validate assumptions leading to the model. The LIBS data were obtained from the echelle spectrograph which is connected with an intensified charge-coupled device (ICCD) sensors (iStar, Andor Technology, DH734-18F 03) [13, 14] and establish the optimal classifier for this type of data. Then, we utilize our model to verify the performance. Note that this specific device is a representative of the current state-of-the-art spectrometers and de facto industry standard. Therefore, the presented approach well generalizes to other similar devices.

**Fig. 1:**
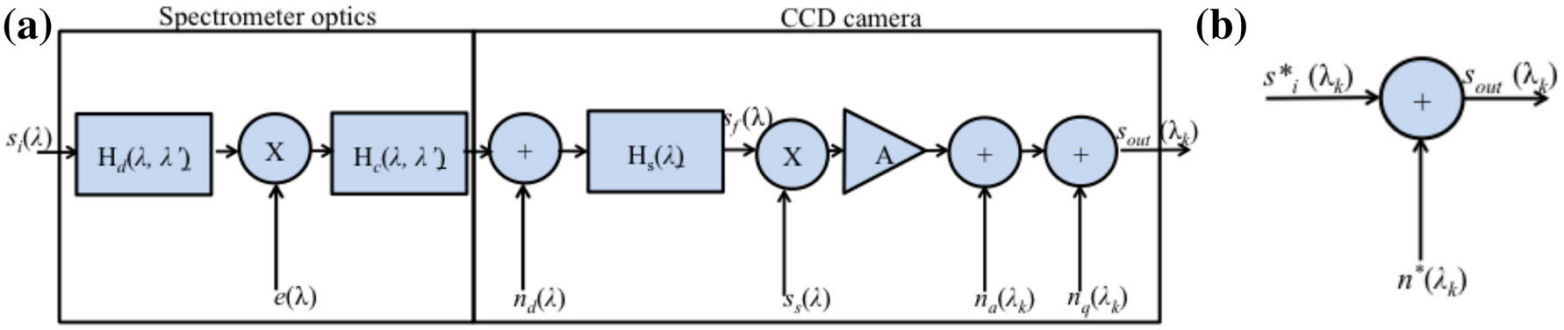
**a** Block diagram of spectrograph; **b** simplified block diagram

## Methodology

### Model of spectroscopy system

**Fig. 2:**
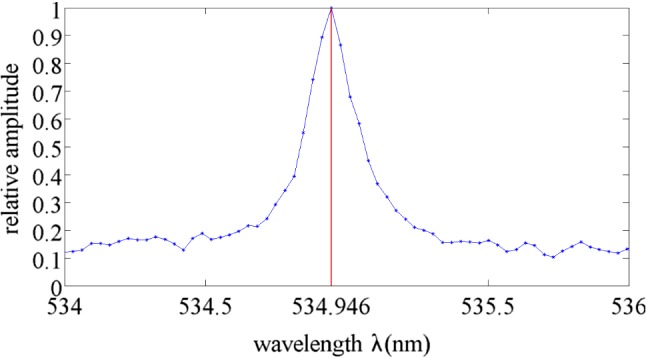
Appearance of spectral line of 534.946 nm of NIST standard reference wafer 612. Shown are effects of spectral line widening due to interference and diffraction at diffraction grating

The block diagram of an Andor Mechelle 5000 spectrograph system based on the echelle grating [13, 14] is shown in Fig. 1a, while Fig. 1b shows the simplified block diagram. The goal of the system is to measure spectrum $s_{i}(\uplambda ), \uplambda \in [ \uplambda _{\min }, \uplambda _{\max }]$ of a light source, where $\uplambda _{\min }$ and $\uplambda _{\max }$ denote minimal and maximal wavelengths of light registered by the system. The spectrograph is modeled as a linear system consisting of spectrometer optics and CCD camera. The following describes role of each block in Fig. 1a. The light from a broadband source passes through diffraction grating, which creates the high dispersion of the wavelength into several different directions. Due to diffraction and interference [14, 15], spectral lines widening phenomena occur, see Fig. 2. The spectral lines widening can be modeled through the following convolution:

1 $$\begin{aligned} s_\mathrm{d}\left( \lambda \right) =\int ^{{\lambda }_{\max }}_{{\lambda }_{\min }}{H_\mathrm{d}\left( \lambda ,\lambda '\right) s_i\left( \lambda '\right) \mathrm{d}\lambda '}, \end{aligned}$$

where, $H_\mathrm{d}\left( \lambda ,\lambda '\right)$ is a wavelength-dependent impulse response of the system.

The intensity of the measured signal is proportional to the echelle efficiency [15, 16] $e(\lambda )$ that is wavelength dependent.

Note that in the echelle spectrometer, high-order diffraction orders are utilized, and the measurements in each order appear as one linear pattern on the detector. The uneven distribution of orders may lead to closely stacking-up orders and cross talk (“ghost line”) [13, 15]. We model cross talk with a linear system with pulse response $H_\mathrm{c}\left( \lambda ,\lambda '\right)$.

The light is converted into electrical signal in a CCD sensor, where the number of electrons at each pixel is proportional to the intensity of the incident light at the pixel. In a CCD sensor, three types of noises exist based on the intensity of photon signal present on CCD pixel [17]. These three noises are: read-out noise (at low light intensities), shot noise (at medium intensities), and fixed pattern noise (at high intensities). The shot noise is a combination of photon noise and dark noise. Photon noise comes from random variation of photon flux from the light source, while the dark noise is created because of the thermal generation of carriers. Fixed pattern noise exists because of the variation of charge created in individual pixels of CCD for photon signal input. Considering the laser signal as medium intensity, the dominating noise source for this case is shot noise, which comes mainly from dark current as the device was operating at room temperature. We assume that component of dark current noise is $$n_\mathrm{d}$$($$\lambda $$) [18]. In the CCD sensor, the signal gets discretized in space (corresponding to discrete wavelength $${\lambda }_k$$) which we model with a low-pass filter $$H_\mathrm{s}\left( \lambda \right) $$ followed by multiplication with a Dirac pulse trail $$s_\mathrm{s}\left( \lambda \right) =\sum ^K_{k=1}{\delta \left( \lambda -{\lambda }_k\right) }$$ (e.g., [19]). The pixel voltages get amplified (A) and quantized. The amplifier introduces the amplifier noise $$n_\mathrm{a}$$($${\lambda }_k)$$. The quantization adds quantization noise $$n_\mathrm{q}$$($${\lambda }_k)$$. The output of the system is therefore the signal $$s_\mathrm{out}$$($${\lambda }_k$$) discretized in the wavelength domain. Due to the linearity of the observed system, it can be simplified as shown in Fig. 1b. The output signal $$s_\mathrm{out}$$$${(\lambda }_k$$) consists of the equivalent input signal $$s^{*}_{i}$$$${(\lambda }_k$$) and additive equivalent noise $$n^{*}$$$${(\lambda }_k$$).

Note that the input signal ${s}_i\ (\lambda _k)$ is proportional to the number of photons with energy $h_\mathrm{c}/\lambda _k$ and hence has a Poisson distribution [20]. Under assumption that ${s}_i (\lambda _i$) and ${s}_i (\lambda _j)$ are independent for $\lambda _i\ne \lambda _j$, since the sum of independent Poisson variables has Poisson distribution [21], ${s}^*_i (\lambda _k)$ has the Poisson distribution which can be approximated as Gaussian when its mean is large enough [22].

Dark current noise $n_\mathrm{d}$($$\lambda )\ $$ is here modeled as Gaussian [23]. The read-out noise $n_\mathrm{a}$(${\lambda }_k)$ consists of thermal (Johnson) noise and 1/*f*—noise (flicker) noise and can also be modeled as Gaussian [24]. Quantization noise $n_\mathrm{q}$(${\lambda }_k)$, on the other hand, has uniform distribution (if the quantizer is not overloaded) and is not correlated with the discretized signal value [25]. We assume that the number of quantization levels is large enough so that the influence of $n_\mathrm{q}({\lambda }_k)$ is small and that $n^*\left( {\lambda }_k\right)$ can also be modeled as Gaussian.

It is known [26] that dark current noise in CCD detectors is spatially uncorrelated (leading to $$E(n_\mathrm{d}({\lambda }_i)n_\mathrm{d}{(}{\lambda }_i))=0,{\lambda }_i\ne {\lambda }_j$$). (Here, *E* denotes expectation.) We assume that the independence of the noise applies to *all* components of $$n^{*}$$$${(\lambda }_k$$), i.e., $$E({n^{*}}({\lambda }_i)n^*{(}{\lambda }_i))=0,{\lambda }_i\ne {\lambda }_j$$.

### Optimal classifier of spectroscopy data

The goal of classification is to distinguish between two hypotheses:

$$\begin{aligned} H_1:s_\mathrm{out}\left( {\lambda }_k\right)= & {} s^*_{i,1}\left( {\lambda }_k\right) +\ n^*\left( {\lambda }_k\right) ,k=1,\dots K\\ H_2:s_\mathrm{out}\left( {\lambda }_k\right)= & {} s^*_{i,2}\left( {\lambda }_k\right) +\ n^*\left( {\lambda }_k\right) ,k=1,\dots ,K \end{aligned}$$

based on observed values of $s_\mathrm{out}\left( {\lambda }_k\right) , k=1,\dots , K.$

Following the discussion in Sect. 2.1, we assume that $s^*_{i,1}\left( {\lambda }_k\right) ,s^*_{i,2}\left( {\lambda }_k\right)$ and $$n^*\left( {\lambda }_k\right) \ $$ are Gaussian. Since the sum of two Gaussian variables is always Gaussian [21], we can write hypotheses in the vector form:

2 $$\begin{aligned} H_1:{{\mathbf{s}}}_\mathrm{out}= & {} {{\mathbf{r}}}_{{1}}, \nonumber \\ H_2:{{\mathbf{s}}}_\mathrm{out}= & {} {{\mathbf{r}}}_{{2}}, \end{aligned}$$

where ${{\mathbf{r}}}_{{1}}$ and ${{\mathbf{r}}}_{{2}}$ are *K*-variate Gaussian vectors. By the Gaussian assumption, a sample ${{\mathbf{s}}}_\mathrm{out}$ has the following conditional probability density function under hypothesis $H_{i}, i=1,2$ [27]:

3 $$\begin{aligned} p\left( {{\mathbf{s}}}_\mathrm{out}|H_i\right) = \frac{1}{{\left( 2\pi \right) }^{K/{\varvec{2}}}{|{\varvec{{\Sigma }}}_i|}^{1/2}}\mathrm{e}^{-\frac{1}{2}{\left( {{{\mathbf{s}}}_\mathrm{out}{\mathbf{-}}{\mathbf{m}}}_i\right) }^{{\mathrm{T}}}{{\varvec{{\Sigma }}}_i}^{-1}\left( {{{\mathbf{s}}}_\mathrm{out}{\mathbf{-}}{\mathbf{m}}}_i\right) },\nonumber \\ \end{aligned}$$

where the mean vectors ${{\mathbf{m}}}_i$ and K*K covariance matrices are defined as:

4 $$\begin{aligned} {{\mathbf{m}}}_i\triangleq & {} E\left( {{\mathbf{r}}}_i\right) , \nonumber \\ {\varvec{{\Sigma }}}_i\triangleq & {} E\left( \left( {{{\mathbf{m}}}_i{\mathbf{-}}{\mathbf{r}}}_i\right) {\left( {{{\mathbf{m}}}_i{\mathbf{-}}{\mathbf{r}}}_i\right) }^{{\mathrm{T}}}\right) , i = 1, 2. \end{aligned}$$

The likelihood ratio test [27] decides between hypotheses based on comparison of the likelihood ratio ${\varvec{{\Lambda }}}\left( {{\mathbf{s}}}_\mathrm{out}\right)$ defined as:

5 $$\begin{aligned} {\varvec{\Lambda }}\left( {{\mathbf{s}}}_\mathrm{out}\right) \triangleq \frac{p\left( {{\mathbf{s}}}_\mathrm{out}|H_2\right) }{p\left( {{\mathbf{s}}}_\mathrm{out}|H_1\right) } \end{aligned}$$

with a threshold $\eta$. If ${\varvec{\Lambda }}\left( {{\mathbf{s}}}_\mathrm{out}\right) > \eta$, it decides hypothesis $H_2$: Otherwise, $H_1$ is decided. The threshold $\eta$ depends on the chosen performance criteria (e.g., minimization of total error as in maximum a posteriori probability test).

By taking logarithm and arranging the terms, from Eq. (5) we obtain the following log-likelihood test, which represents the optimal classifier under the Gaussian assumption:

1. Calculate:
  6 $$\begin{aligned} l\left( {{\mathbf{s}}}_\mathrm{out}\right) ={{\mathbf{s}}}^\mathrm{T}_\mathrm{out}{\mathbf{A}}\varvec{\ }{{\mathbf{s}}}_\mathbf{out }{+}{{\mathbf{b}}}^\mathrm{T}{{\mathbf{s}}}_\mathbf{out } -\gamma \end{aligned}$$
  where
  6a $$\begin{aligned} {\mathbf{A}}&\triangleq \frac{1}{2}\left( {\varvec{{\Sigma }}}^{-1}_1-{\varvec{{\Sigma }}}^{-1}_2\right) \nonumber \\ {\mathbf{b}}&\triangleq {\varvec{{\Sigma }}}^{-1}_2{{\mathbf{m}}}_2-{\varvec{{\Sigma }}}^{-1}_1{{\mathbf{m}}}_1 \nonumber \\ \gamma&\triangleq \ln \ \eta +\frac{1}{2}\left( {{\ln } \left| {\varvec{{\Sigma }}}_2\right| \ }-{{\ln } \left| {\varvec{{\Sigma }}}_1\right| }\right. \nonumber \\&\quad \left. {+{{\mathbf{m}}}^\mathrm{T}_2{\varvec{{\Sigma }}}^{-1}_2{{\mathbf{m}}}_2-{{{\mathbf{m}}}^\mathrm{T}_1\varvec{{\Sigma }}}^{-1}_1{{\mathbf{m}}}_1\ }\right) ; \end{aligned}$$
2. If $l\left( {{\mathbf{s}}}_\mathrm{out}\right) > 0$, decide $H_2$; otherwise, decide $H_1$.

Note that when the statistical parameters of output signal, Eq. (4), are known, log-likelihood test, Eq. (6), results in decision boundary *quadratic* in terms of the observed output vector of the system.

From machine learning point of view, the algorithm of automatic classifier can be specified as:

1. Estimate mean vectors ${\mathbf{m}}_1$, ${\mathbf{m}}_2$ and covariance matrices $\varvec{{\Sigma }}_1^{-1}$, $\varvec{{\Sigma }}_2^{-1}$ , Eq. (4), from K-dimensional observations data belonging to classes 1 and 2 (and corresponding to ${{H}}_1$, ${{H}}_2$);
2. Choose threshold $$\eta $$;
3. Calculate matrix **A**, vector **b**, and scalar $\gamma$, Eq. (6a);
4. For each sample $s_\mathrm{out}$, calculate $l\left( {{\mathbf{s}}}_\mathrm{out}\right)$, Eq. (6), and perform classification.

Note that, from Eq. (6), the optimal classifier results in quadratic decision boundary ${{\mathbf{s}}}^\mathrm{T}_\mathrm{out}{\mathbf{A}}\varvec{\ }{{\mathbf{s}}}_\mathbf{out }+{{\mathbf{b}}}^\mathrm{T}{{\mathbf{s}}}_\mathbf{out } = \gamma$.

Observe that the parameters of the decision boundary are not directly related to LIBS wavelengths (but are related to measurements obtained from the spectrometer). In other words, the wavelengths themselves are not input to the model.

Assuming the availability of a sufficiently large number $({n}>{K}+1)$ of experimental realizations, means and the invertible covariance matrices, Eq. (4), can be estimated from experimental data [27]. The estimates can be subsequently plugged into Eqs. (6)–(6a). Alternatively, an approximately optimal classifier can be obtained using support vector machines (SVM) [28] with the following polynomial kernel:

7 $$\begin{aligned} \kappa \left( {\mathbf{x}},{\mathbf{y}}\right) {=\left( {{\mathbf{x}}}^\mathrm{T}{\mathbf{y}}+1\right) }^2, \end{aligned}$$

where, **x** and **y** are *K*-dimensional feature vectors.

Note that in addition to original features, $s_\mathrm{out} (\lambda _k), k=1, \dots , K$, the classifier can be applied on *linearly transformed* features $y_j = f_j(s_\mathrm{out} (\lambda _1 ), \dots , s_\mathrm{out} (\lambda _K ),j = 1, \dots , K'$ where $K'\le K$. Such features can, e.g., be obtained using principal component analysis (PCA) [29]. In such a case, assuming Eq. (3) holds, the transformed features $y_j$ also have normal distribution [30]. Hence, for classification of spectroscopy data transformed using PCA, the quadratic classifier is also optimal.

### Experimental setup

**Fig. 3:**
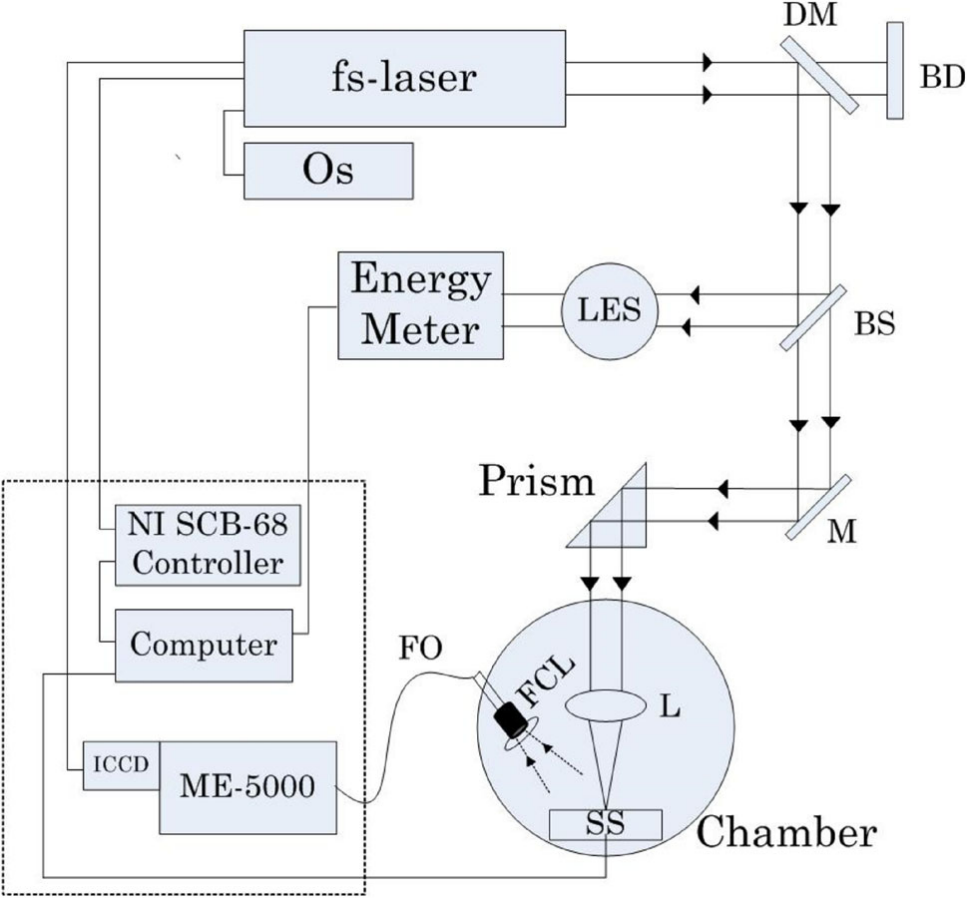
Block diagram of the LIBS system used to collect the data

We utilized Andor Mechelle ME5000 spectrograph with an ICCD camera (iStar, Andor Technology, DH734-18F 03), see Fig. 3. The following parameters are from the technical specifications of the spectrograph and correspond to usual spectroscopy practice. The spectral resolution (the ratio between the wavelength and the smallest difference of wavelengths that can be resolved) was $R=$ 4000 corresponding to 4 pixels FWHM [31]. The total number of channels was 26,040. The wavelength range was 199.04–974.83 nm. The spectrometer uses diffraction orders ${m} =$ 21–100. The grating had 52.13 line/mm with grating constant $d\approx$ 5–30 $\upmu$m, blazed at 32.35 degree. The spectra were collected 50 ns after the laser pulse with integration time of 700 $\upmu$s by an on-board digital delay generator (DDG) of the spectrograph. The CCD was kept at a stable temperature at − 10 $^{\circ }$C using a thermoelectric (TE) cooler of the spectrograph to reduce dark signal (see Sect. 2.1). To excite plasma in LIBS [32], a broadband CPA-Series Ti: Sapphire ultra-short laser (Clark-MXR, Inc., Model: 2210) generating 150-fs-long pulses operating at the wavelength of 775 nm was used. For experiments with dark signal, the laser beam was blocked by a nontransparent barrier. This way, we capture only the system’s noise.

## Experimental results

### Experiments of “dark signal”

To quantify characteristics of CCD sensor, 1000 dark spectra were acquired with no source of light incident to the sensor. The goal was to test the following hypotheses:

$\hbox {H}_{01}$: $s_\mathrm{out}$($\lambda _{k}$) follows Gaussian distribution, $\lambda _{k}\in$ [200.33 nm, 909.45 nm].

Note that outside this range the spectrometer provided signals equal to zero for all realizations. The total range of considered wavelengths included 24,650 discrete values.

$\hbox {H}_{02}$: $s_\mathrm{out} (\lambda _i)$, $s_\mathrm{out}(\lambda _j)$ are uncorrelated when $\lambda _i {\ne }\lambda _j$.

To test $\hbox {H}_{01}$, we used Kolmogorov-Smirnov [33] and Lilliefors test [34]. In addition, we computed skewness and kurtosis for observations $s_{\mathrm{out}, i} (\lambda _k), i=1, \ldots , 1000$ at each wavelength. The Kolmogorov-Smirnov test indicated that $\hbox {H}_{01}$ can be rejected at 25 out of 24,650 wavelengths at the significance level $\alpha =0.05$. The Lilliefors test indicated that $\hbox {H}_{01}$ can be rejected at 1622, 366, and 212 wavelengths, with $\alpha =0.05$, $\alpha =0.01$, and $\alpha =0.005$, respectively.

For the 25 wavelengths where $\hbox {H}_{01}$ was rejected using the Kolmogorov-Smirnov test, we visually examined the histograms of 1000 realizations. For wavelengths 211.9 nm, 228.19 nm, 303.82 nm, the histograms indicated that the distribution of $s_\mathrm{out} (\lambda _k)$ may be bimodal. For the other wavelengths, the histograms indicate the presence of obvious outliers. These outliers (the maximal values) corresponded to eight realizations that were subsequently removed from the dataset.

**Fig. 4:**
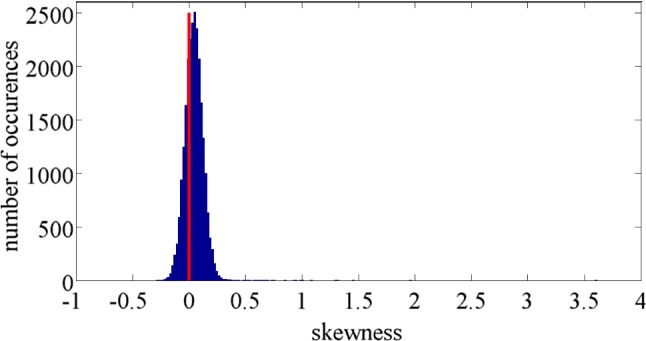
Histogram of estimated skewness of dark signal at all observed wavelengths. The skewness of zero, characteristic for Gaussian distribution, is denoted by red line

**Fig. 5:**
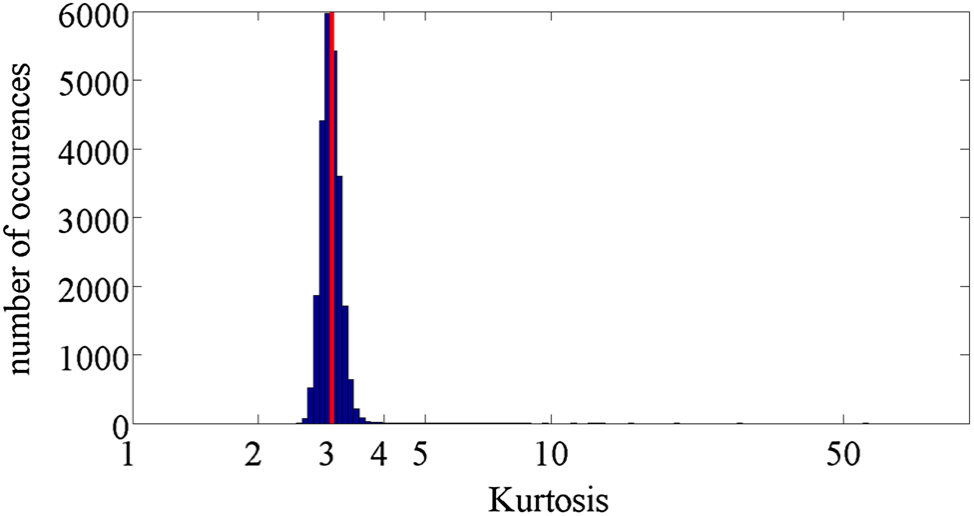
Histogram of estimated kurtosis of dark signal at all observed wavelengths. The kurtosis of 3, characteristic for Gaussian distribution, is denoted by red line

The skewness and kurtosis [35] were calculated for each $s_\mathrm{out}(\lambda _k)$ using the remaining 992 realizations. Figures 4 and 5 show histograms of the obtained skewness and kurtosis.

**Fig. 6:**
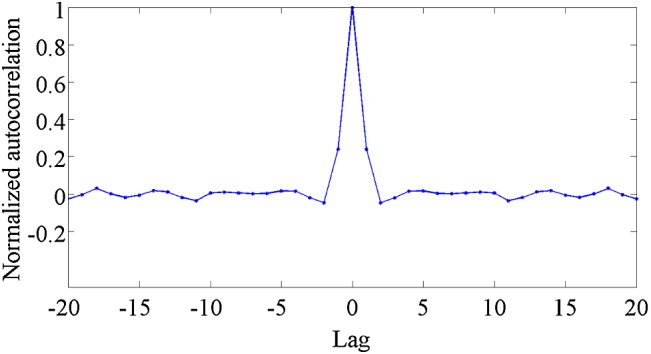
Estimated normalized autocorrelation of “dark signal”

To test $\hbox {H}_{02}$, we estimated normalized sample autocorrelation [36] of signals $s_\mathrm{out} (\lambda {}_k)$ in the domain of discretized wavelengths $\lambda _k$, $k=1, \ldots , K$. First, for each spectral order *m*, we determined discrete wavelengths $\lambda _{m,1}<\lambda _{m,2}<\cdots<\lambda _k<\cdots \lambda _{{m,\ m}_k}$ satisfying $m=$ round $\left( \frac{20,139}{\lambda _k} \right)$ (where $\lambda _k$ is given in nanometers) [31]. Then, we computed sample autocorrelations $r_m (l)$ for signals $s\left( {\lambda }_{m,1}\right) ,\dots ,s \left( {\lambda }_{{m,m}_k}\right)$ where the signals in each realizations were normalized to have the zero mean. Finally, we averaged normalized correlations $r_m(l)/r_m(0)$ for $m=21, \dots 100$. The averaged normalized correlations for lags $-20, \dots , 20$ are shown in Fig. 6.

### Experiments with standardized data

We measured laser-induced breakdown spectroscopy (LIBS) spectra [32] of NIST standardized glass. According to National Institute of Standards & Technology (NIST), the nominal composition of the standard reference wafer 612 used in this work is 72% $\hbox {SiO}{}_{2}$, 12% CaO, 14% $\hbox {Na}{}_{2}$O, and 2% $\hbox {Al}{}_{2}\hbox {O}{}_{3}$. Total 61 trace elements are included in the glass support matrix. The reference wafer is specifically intended for evaluating analytical techniques used to determine trace elements in inorganic matrices [37]. For a sample of NIST standardized glass, we performed 150 realizations of spectra. This was repeated seven times, for seven different samples, resulting in total of $n=1050$ spectral realizations. We repeated procedure indicated in Sect. 3.1 to test $H_{01}$ (Gaussianity). Table 1 indicates spectral ranges where $H_{01}$ cannot be rejected using different tests and significance levels.

We also computed principal components of the NIST standardized glass data. This resulted in total of $1049 (=n-1)$ principal components. Out of the first 100 principal components, components 5, 6, 8, 9, 11, 100 were identified to have Gaussian distribution using both Kolmogorov-Smirnov test with $\alpha = 0.05$ and the Lilliefors test with $\alpha = 0.005$.

## Discussion

### Experiments of “dark signal”

Kolmogorov-Smirnov (KS) test indicates that the hypothesis of Gaussian distribution of “dark signal” holds for almost all wavelengths. (The $\hbox {H}{}_{01}$ could not be rejected even with very large significance level $\alpha$.) Lilliefors test indicates that the number of wavelengths where the Gaussian distribution holds is smaller than the number indicated by the KS test. It is shown [38] that KS test tends to be inferior to Lilliefors test when the parameters of the Gaussian distribution are unknown. In such a case, the Lilliefors test has higher power (smaller probability of false acceptance of $\hbox {H}{}_{01}$). Hence, there is no wonder that the number of wavelengths where $\hbox {H}{}_{01 }$ is rejected (Gaussian distribution is not satisfied) is larger with the Lilliefors test than with the Kolmogorov-Smirnov test (for the same $\alpha =0.05$).

Histogram of estimated skewness, Fig. 4, indicates that the mode of the skewness is slightly larger than 0 (the skewness of normal distribution). From Fig. 5, the mode of kurtosis is around 3 (the kurtosis of the normal distribution). Based on these results, it can be concluded that the probability distribution of CCD noise is approximately normal for a large percentage of wavelengths.

The estimated normalized autocorrelation of “dark signal,” Fig. 6, indicates that the dark noise samples are observably correlated only with the samples at adjacent wavelengths. Hence, $\hbox {H}{}_{02}$ cannot be completely accepted. The assumption of whiteness ($\hbox {H}{}_{02}$), however, is *not* needed in our model.

### Experiments with NIST glass

The results of Kolmogorov-Smirnov and Lilliefors tests on LIBS spectra of standardized NIST glass indicate that the distributions of signals $s_\mathrm{out}(\lambda _k)$ can be considered approximately Gaussian for a large range of $\lambda _k$ (notably, when $\lambda _k \in$ [320 nm, 581 nm] for all attempted tests). Due to observed Gaussian distribution of the dark signal, this leads to conclusion that $s_i(\lambda _k)$ in the considered case have approximate Gaussian distribution in this range of wavelengths. Furthermore, almost all low-order principal components of the data (that are of practical importance for classification, see e.g., [39]) also have Gaussian distribution.

**Table 1:** Spectral ranges where the hypothesis of Gaussianity of LIBS spectra of NIST standardized glass cannot be rejected

Test	Significance level ($\alpha$)	Spectral range (nm)
Kolmogorov-Smirnov	0.05	306–581
Lilliefors	0.05	320–721
Lilliefors	0.005	311–749

### Applicability of the optimal classifier

Classification of LIBS data has been an active area of research. Automatic classification has been attempted on a variety of domains including mineralogy (classification of sedimentary ores [40], quartz samples [41], material science [42], botany [43], homeland security [44], and planetology [45])

The optimal classifier presented in the paper is relatively simple. (Classification is performed by computing a quadratic function of observed discrete spectral components.) This highly contrasts with sophisticated and complex classifiers previously attempted in the literature [7, 9–11, 42].

The usage of the proposed classifier can be validated using a cross-validation technique [46]. An available dataset is split into *k* disjoint subsets of approximately equal size. The classifier is trained using $k-1$ subsets, and the classification accuracy is evaluated on the remaining subset. The procedure is repeated until all subsets are utilized for the evaluation of the classifier. This way, assumptions of the optimal classifier can be indirectly validated on particular data. Using this approach, in [39] we demonstrated that a simplified version of the optimal classifier discussed in this study is capable of providing high classification accuracies ($>90\%$) when a sufficiently large number of principal components are utilized to perform multi-class classification of LIBS data of four proteins diluted in phosphate-buffered saline solution (bovine serum albumin, osteopontin, leptin, insulin-like growth factor II). This result is in agreement with the findings shown in this study that the principal components predominantly have Gaussian distribution. Note that the applicability of the optimal classifiers depends on our ability to estimate statistical parameters of output signal, Eq. (4), specifically the covariance matrices $\varvec{{\Sigma }}_i$. If the number of samples per class is small in comparison with the dimension of covariance matrices, additional assumptions about the structure of the matrices are needed (e.g., in [39], we assumed matrix diagonality). Alternatively, the dimensionality of the correlation matrices can be reduced if a number of considered wavelengths are decreased by methods of feature selection (e.g., [35]).

A practitioner may be interested what are the features that are responsible for successful classification. Answer to this depends on which particular classification problem we try to solve (e.g., classification of various compounds, the presence of elements). If feature selection [47] is used for dimensionality reduction, the wavelengths corresponding to selected spectral lines indicate which spectral lines are responsible for building a classification model. In contrast, if feature extraction methods are used [39], the loads (weight factors utilized to calculate principal components) may provide indication of relative feature importance.

By employing support vector machines (SVMs), we can estimate a hypothesis drawn from the function class of polynomials that both separates the data and achieves the maximum margin. SVMs carry the benefit of the descriptive power afforded by models with large degrees of freedom while incurring the complexity (VC dimension) of a relatively small number of support vectors. In the SVM formulation, through “the kernel trick,” a transformation of input space is implemented through the definition of its inner product over the set of in-sample data points. The kernel can be thought of as a transformation of the input space to a high-dimensional representational space (or feature space). This also has the effect of further reducing the computational burden by avoiding computation of inner products in a high-dimensional feature space. The model linear in the feature space induced by the kernel represents the equivalent nonlinear model in the input space. Note that classification of spectroscopy data using SVMs was successfully attempted in [48]. Note, however, that for large *K*, the actual estimation of model coefficients may require excessive computational power.

Equation (6) represents the optimal classifier if the assumption of Gaussian distribution holds. Our experimental results indicate that the Gaussian distribution holds for noise and for *specific* spectroscopy signal in a range of wavelengths. In reality, signals $$s_i\left( \lambda \right) $$ have Poisson distribution. If distributions of the signals $$s_i\left( \lambda \right) $$ at two different wavelengths are *independent*, the signal components $$\ s_f\left( \lambda \right) $$ before sampling will also have Poisson distribution that can be approximated by Gaussian. However, if the distributions are *dependent*, $$s_f\left( \lambda \right) $$ as an integral of dependent Poisson variables does not *have* to be Poisson random variable [49]. Further, $$n{}^{*}$$$${(\lambda }_k$$) may not be Gaussian random variables. If the assumptions of Gaussian distribution are not satisfied, techniques of classification of non-Gaussian signals in generalized (non-Gaussian) noise need to be considered [50–52].

Finally, the optimal classifier presented in this paper assumes that the signal flow in the spectrometer can be represented by *linear* systems $$H_\mathrm{d}\left( \lambda ,\lambda '\right) ,H_\mathrm{c}\left( \lambda ,\lambda '\right) \ $$and $H_\mathrm{s}\left( \lambda \right)$. Further research is needed to develop the optimal classifier if the assumption of linearity does not hold.

## Conclusions

We discussed the optimal classifier for a signal acquisition model in echelle spectrograph and validated model assumptions in a case of specific LIBS signal. We indicated that the optimal classifier has a quadratic decision boundary and can be approximated using SVMs with a quadratic kernel. The optimal classifier can function with features obtained using a feature selection or feature extraction (principal component analysis) method. Experimental results indicate that in the considered case, the assumptions of Gaussianity hold. Work in progress includes development of the optimal classifier when assumptions of Gaussianity and linearity are relaxed.
